# Predicting nodal response to neoadjuvant treatment in breast cancer with core biopsy biomarkers of tumor microenvironment using data mining

**DOI:** 10.1007/s10549-024-07539-9

**Published:** 2024-11-04

**Authors:** Nina Pislar, Gorana Gasljevic, Erika Matos, Gasper Pilko, Janez Zgajnar, Andraz Perhavec

**Affiliations:** 1 Department of Surgical Oncology, Institute of Oncology Ljubljana, Ljubljana, Slovenia; 2 Faculty of Medicine, University of Ljubljana, Ljubljana, Slovenia; 3 Department of Pathology, Institute of Oncology Ljubljana, Ljubljana, Slovenia; 4 Faculty of Medicine, University of Maribor, Maribor, Slovenia; 5 Department of Medical Oncology, Institute of Oncology Ljubljana, Ljubljana, Slovenia

**Keywords:** Breast cancer, Neoadjuvant, Nodal response, Prediction model, Tumor microenvironment

## Abstract

**Purpose:**

To generate a model for predicting nodal response to neoadjuvant systemic treatment (NAST) in biopsy-proven node-positive breast cancer patients (cN+) that incorporates tumor microenvironment (TME) characteristics and could be used for planning the axillary surgical staging procedure.

**Methods:**

Clinical and pathologic features were retrospectively collected for 437 patients. Core biopsy (CB) samples were reviewed for stromal content and tumor-infiltrating lymphocytes (TIL). Orange Datamining Toolbox was used for model generation and assessment.

**Results:**

151/437 (34.6%) patients achieved nodal pCR (ypN0). The following 5 variables were included in the prediction model: ER, Her-2, grade, stroma content and TILs. After stratified tenfold cross-validation, the logistic regression algorithm achieved and area under the ROC curve (AUC) of 0.86 and F1 score of 0.72. Nomogram was used for visualization.

**Conclusions:**

We developed a clinical tool to predict nodal pCR for cN+ patients after NAST that includes biomarkers of TME and achieves an AUC of 0.86 after tenfold cross-validation.

**Supplementary Information:**

The online version contains supplementary material available at 10.1007/s10549-024-07539-9.

## Introduction

Neoadjuvant systemic treatment (NAST) has become a standard treatment strategy for patients with stage II-III breast cancer [1]. It allows downstaging of the disease and assessment of response to therapy, which facilitates post-neoadjuvant treatment planning. Response to NAST is not only a favorable prognostic factor, but also allows for less aggressive surgical management of the axilla in this setting [2, 3].

For initially node-positive patients (cN+), axillary lymph node dissection (ALND) used to be the standard surgical procedure. However, in the absence of nodal metastases after NAST, sentinel lymph node biopsy (SLNB) with 3 or more nodes retrieved or other limited surgical options have been proven as feasible alternatives with acceptably low false-negative rates [4–7]. Performing SLNB alone did not translate into worse outcome even after 10 years of follow-up [8].

Pathologic complete response (pCR) in the axilla is expected in up to 70% of cases, but not all patients are successfully spared ALND [9]. Although imaging has improved the preoperative nodal assessment compared to clinical assessment alone, its conventional use is still inferior to surgical staging [10]. With an accuracy of up to 70%, axillary ultrasound may lead to an incorrect surgical strategy in about one third of cases [11].

Nodal pCR is associated with the biological subtype of the tumor as well as other clinical and pathologic characteristics [12, 13]. In addition to tumor cell characteristics, the tumor microenvironment (TME) has gained attention in recent years to investigate both prognostic and predictive value in several cancers [14–16]. Tumor-infiltrating lymphocytes (TIL) and the tumor stroma are major constituents of TME and allow for an overall stromal landscape assessment [17, 18]. While the prognostic and predictive value of TILs in breast cancer has been thoroughly studied [19, 20], the content of tumor stroma has been shown to be not only a prognostic but also a predictive biomarker [21, 22].

The aim of the present study was to use data mining to create a model for predicting nodal response to NAST in cN+ patients that incorporates TME characteristics and could be used to optimize surgical planning.

## Methods

### Data collection

The study was approved by the national ethics committee (approval number 0120–178/2022/3). Due to the retrospective nature of the study, informed consent was waived. We retrospectively reviewed the records of patients diagnosed with breast cancer at our institution between 2008 and 2021. Female patients who were treated with NAST followed by surgery were eligible for the study. Patients with inflammatory breast cancer, primary metastatic breast cancer, bilateral breast cancer, synchronous malignancy at other site, history of invasive or in situ carcinoma of the breast, or breast cancer diagnosed during pregnancy were excluded. Only node-positive (cN+) patients were included in the study, which was defined as biopsy-proven nodal involvement at presentation.

The following clinical and pathologic characteristics were recorded retrospectively: Age at diagnosis, menopausal status, tumor and nodal stage at diagnosis (according to TNM), pre-treatment core-needle biopsy (CB) tumor characteristics: ER %, PR %, Her-2 status, Ki-67%, grade, histological subtype, radiological response on breast imaging after NAST and axillary ultrasound findings after NAST. Subtype was determined based on CB characteristics.

### Axillary staging procedure

We employ dual-tracer technique with a recommended sampling of at least 3 SLNs for patients that had biopsy-proven node-positive disease before neoadjuvant treatment, as previously described [23].

Overall, 342/437 (78.3%) patients ultimately underwent ALND and the remaining 95/437 patients (21.7%) had sentinel lymph node biopsy (SNLB) only. Of these, 71 had no evidence of disease in the SLNs (neither ITC, micro- nor macrometastases). If the patient was rendered clinically and radiologically (using axillary US) node-negative after treatment, they were planned for SLNB. If there was a high suspicion of residual disease, they were proceeded directly to ALND. However, when less than 3 SLNs were retrieved, the patients would often undergo ALND directly.

### Core biopsy revision

Because TILs and tumor stroma are not routinely reported on CB reports, all pre-treatment hematoxylin–eosin (H&E) slides were reviewed by an experienced breast pathologist.

We had access to a single core biopsy in 30% of cases. For the remaining, multiple cores were available, and we followed the approach outlined by Le et al. This involved screening all available cores to identify the most stroma-rich area of the specimen. The selected cores needed to have invasive tumor cells present at both ends of the specimen, as well as along the side margins, or throughout the entire specimen. The tumor stroma ratio (TSR) was evaluated by tenfold percentage of stroma content.

While 50% was established as the cut-off value for stroma-rich vs. stroma-poor tumors in resection samples, a cut-off value for CB specimen that classifies stroma-rich vs. stroma-poor tumors with optimal specificity and sensitivity (maximal Youden index) was set at 40% [24, 25].

TILs were evaluated on CB and reported as percentage values in accordance with previously published reports [26]. For analysis, TILs were considered as three predefined groups of low (0–10%), intermediate (11–59%) and high TILs (≥ 60%) [20].

The primary end-point was axillary pCR, defined as ypN0 in the final histopathology report.

### Statistical analysis and data mining

Clinical and pathologic features were reported as absolute and relative frequencies. Features were compared between patients who achieved nodal pCR and those who did not using the Pearson chi-square test in IBM SPSS Statistics (IBM corp., Armonk, NY, USA).

The Orange Datamining Toolbox (Laboratory for bioinformatics, University of Ljubljana, Slovenia) was used for model generation [27].

The five most influential features were selected by backward elimination. Naïve Bayes, logistic regression, neural network, k-nearest neighbors and random forest algorithms were used to train the prediction model:

- Naive Bayes: a probabilistic model that is simple yet robust and offers valuable insights into effects of attributes on class probabilities. Its outputs can be visualized using nomograms for clear, intuitive support tools, for example in clinical decision-making process.
- Logistic Regression: we included this for its simplicity and interpretability. It is particularly useful for understanding the contribution of individual predictors in binary classification tasks like ours.
- k-Nearest neighbors (kNN): a non-parametric model that captures non-linear relationships and serves as a contrast to parametric methods. It predicts outcomes based on similarity by measuring distance between data points.
- Random forest: an ensemble method that builds multiple decision trees using random subsets of data and features, with predictions based on majority voting. While highly accurate, Random Forests can be less interpretable due to the complexity of multiple trees. They are useful in handling high-dimensional data and are robust against overfitting.
- Neural Networks: modeled after biological neural networks, they are highly effective for complex supervised learning tasks such as disease prediction. They consist of layers of interconnected nodes (neurons) that assign weights to inputs, adjusting through backpropagation. When multiple layers are used, this becomes "deep learning," which is particularly useful for complex modeling problems.

Stratified tenfold cross-validation was used for model validation. The dataset was divided into ten random subsets (with preserving class distribution). The model was trained and evaluated ten times, each time using a different fold as the testing set and the remaining nine-folds as training set. Cross-validation helps in mitigating bias by using different subsets for training and testing in each iteration, ensuring that the model is exposed to various parts of the dataset. It also helps control variance by averaging the performance over multiple runs, making the evaluation more stable and less sensitive to the particularities of a single random split (e.g., manually splitting the data in a training and testing subset in advance). This is crucial for obtaining a robust estimate of the model's generalization performance across different data instances. Since the dataset is not extremely large, it also maximizes the use of all available data for both training and testing purposes, contributing to a more robust assessment of the model's capabilities.

The area under the ROC curve (AUC), accuracy, sensitivity, specificity and F1 score were used to evaluate the performance of the models. The F1 score is a measure of accuracy that combines precision and recall. It is calculated as follows: F1 = ((precision x recall)/(precision + recall)) × 2. A nomogram was used for visualization [28].

The study is reported according to REMARK guidelines [29].

## Results

Overall, 437 cN+ eligible patients were identified. 151/437 (34.6%) achieved nodal pCR (ypN0). Patients’ clinical and pathologic characteristics are summarized in Table 1.

**Table 1:** Clinical and pathologic characteristics

N = 437	Nodal response		p-value
	pCR N = 151	no pCR N = 286	
*Age*			
≤ 50	56 (32.0%)	119 (68.0%)	0.359
> 50	95 (36.3%)	167 (63.7%)	
*Menopausal status*			
Pre-/perimenopausal	93 (37.2%)	157 (62.8%)	0.219
Postmenopausal	56 (31.5%)	122 (68.5%)	
Missing	2	7	
*cT stage*			
cT1-2	126 (36.7%)	217 (63.3%)	0.067
cT3-4	25 (26.6%)	69 (73.4%)	
*cN stage*			
cN0	30 (42.8%)	40 (57.2%)	0.101
cN1-3	118 (32.7%)	243 (67.3%)	
Missing	4	2	
*Subtype*			
HR + Her-2–	30 (13.6%)	191 (86.4%)	< 0.001
HR + Her-2+	52 (59.8%)	35 (40.2%)	
HR– Her-2+	36 (76.6%)	11 (23.4%)	
HR– Her-2–	33 (40.2%)	49 (59.8%)	
*Histology*			
Ductal	147 (35.9%)	263 (64.1%)	0.071
Lobular	1 (5.9%)	16 (94.1%)	
Mixed	2 (40.0%)	3 (60.0%)	
Other	1 (20.0%)	4 (80.0%)	
*ER*			
Positive	81 (26.5%)	225 (73.5%)	< 0.001
Negative	70 (53.4%)	61 (46.6%)	
*PR*			
Positive	47 (21.3%)	174 (78.7%)	< 0.001
Negative	104 (48.1%)	112 (51.9%)	
*Her-2*			
Positive	88 (65.7%)	46 (34.3%)	< 0.001
Negative	63 (20.8%)	240 (79.2%)	
*Ki-67%*			
< 20	8 (16.0%)	42 (84.0%)	0.003
≥ 20	143 (37.0%)	244 (63.0%)	
*Grade*			
Low/intermediate	37 (20.2%)	146 (79.8%)	< 0.001
High	108 (44.3%)	136 (55.7%)	
Missing	6	4	
*Stroma content*			
≤ 50%	78 (45.3%)	94 (54.7%)	< 0.001
> 50%	73 (27.5%)	192 (72.5%)	
Missing			
*TIL*			
Low	76 (26.2%)	214 (73.8%)	< 0.001
Intermediate	45 (45.0%)	55 (55.0%)	
High	27 (65.9%)	14 (34.1%)	
Missing	3	3	
*Radiological response on breast MRI*			
Complete response	41 (58.6%)	29 (41.5%)	< 0.001
Residual tumor	32 (26.9%)	87 (73.1%)	
Not performed	78	170	
*Axillary US after NAST*			
Negative	69 (38.5%)	110 (61.5%)	0.337
Suspicious/positive	20 (30.8%)	45 (69.2%)	
Not performed	62	131	

*ER* estrogen receptor, *Her-2* human epidermal growth factor receptor-2, *HR* hormone receptor, *MRI* magnetic resonance imaging, *NAST* neoadjuvant systemic treatment, *US* ultrasound, *pCR* pathologic complete response, *PR* progesterone receptor, *TIL* tumor-infiltrating lymphocytes

**Fig. 1:**
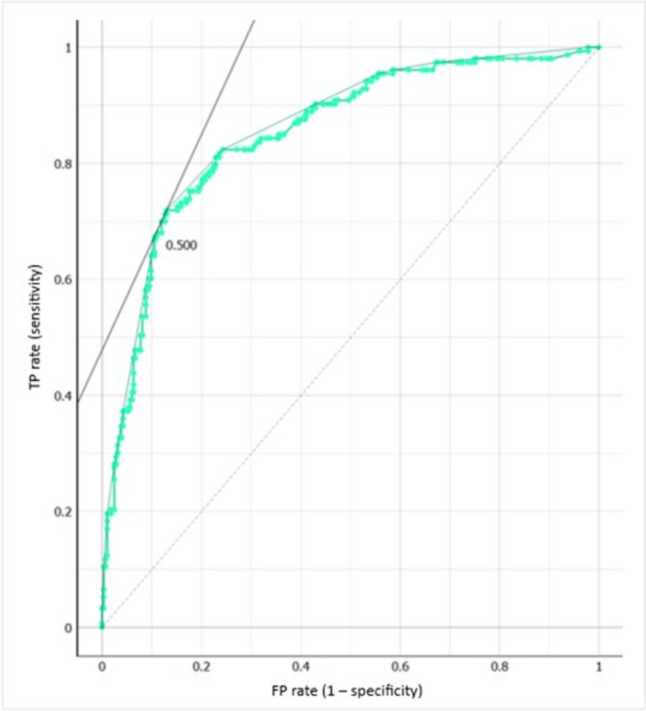
Receiver operating characteristic (ROC) curve. *FP* false positive, *TP* true positive

**Fig. 2:**
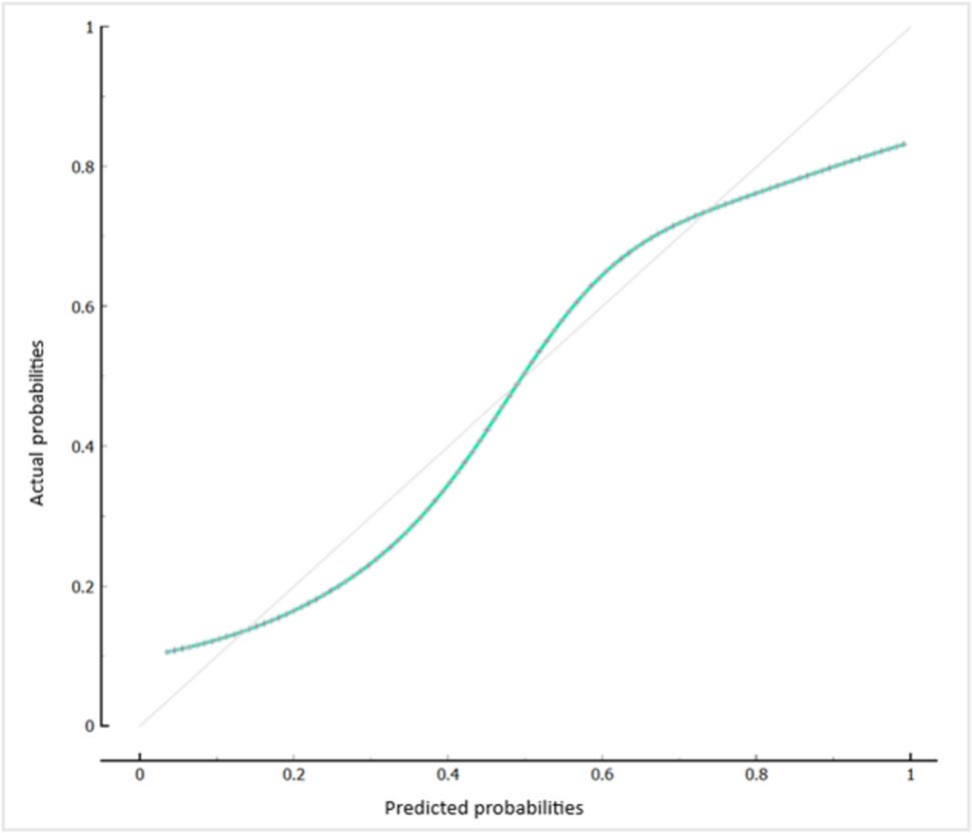
Calibration plot

Features with more than 25% missing values were excluded. After feature reduction, the following 5 variables were included in the model: ER, Her2, grade, stroma content and TILs. Stratified tenfold cross-validation was used for internal validation. The performance of the models for predicting pCR is evaluated in Table 2. The logistic regression model (with ridge regularization) showed the best performance: AUC 0.86, F1 0.72. The ROC curve and calibration curve are shown in Figs. 1 and 2, respectively.

**Table 2:** Models' characteristics

Model	AUC	accuracy	sensitivity	specificity	F1
Naïve Bayes	0.85	0.78	0.65	0.84	0.67
Logistic regression	0.86	0.81	0.68	0.88	0.72
KNN	0.79	0.74	0.56	0.85	0.60
Neural Network	0.86	0.79	0.65	0.87	0.69
Random Forest	0.79	0.76	0.61	0.84	0.64

*AUC* area under the ROC curve, *kNN* k-nearest neighbors

**Fig. 3:**
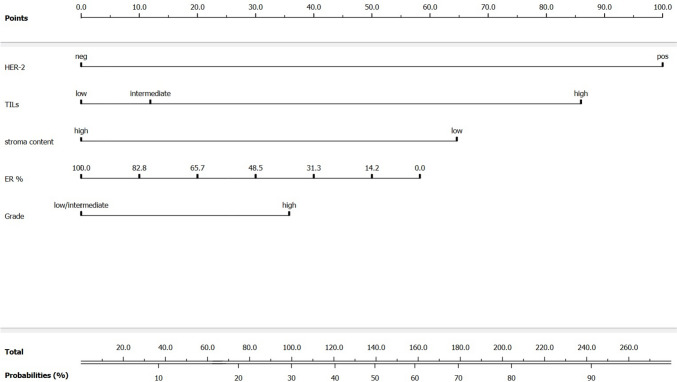
Nomogram. ER-estrogen receptor, HER-2-human epidermal growth factor receptor-2, TIL-tumor-infiltrating lymphocytes

A nomogram was created for visualization (Fig. 3).

## Discussion

Using machine learning methods, we developed a clinical tool to predict nodal pCR in biopsy-proven cN+ patients who underwent NAST. Our model achieved an AUC of 0.86. TILs, Her-2 status, stromal content, ER status and tumor grade were included in the model.

It is worth noting that several prediction models have been previously proposed to estimate nodal pCR rates, taking into account different clinical and pathologic characteristics. Corsi et al. constructed a nomogram on a cohort of nearly 2000 patients; although their model yielded an AUC of 0.77, which is considerably lower compared to our tool, the AUC remained at 0.77 after validation on external cohort [30]. Independent predictors included breast cancer subtype, clinical response in the breast, Ki-67 status and tumor grade. Similarly, in the prediction model developed by Guo et al. Her-2 status, ER expression level, response in the breast, and clinical stage at diagnosis were recognized as independent predictors of nodal pCR in hormone receptor positive breast cancer patients [31]. Kim et al. also developed a nomogram on a cohort of 415 patients with a comparable pCR rate to our cohort [32]. After validation on an independent testing cohort, the model yielded an AUC of 0.8. Of note, all patients underwent ALND to determine the final nodal status, which would no longer be acceptable in clinical practice.

We developed the model using several commonly used algorithms and selected logistic regression as the model with the best performance characteristics. The high and comparable performance across different algorithms further strengthens the predictive value of the input parameters. In addition, Orange facilitates visualization of the logistic regression model through a nomogram, which is a valuable asset for the clinical setting [33].

To reiterate, high TILs, Her-2-positive status, low stromal content, ER below 85% and high grade were predictors of nodal pCR in our model. It has been previously reported that stromal content of CB can predict response in the breast in Her-2-negative tumors by assessing the reduction of tumor cellularity [34]. To our knowledge, this is the first study to report stroma content from pre-treatment CB of primary breast cancer as a predictor of nodal pCR in cytologically proven cN+ patients. In addition, our model includes TILs, which along with TSR provide a comprehensive assessment of TME [17, 35].

TILs serve as a surrogate biomarker of anti-tumor T-cell-mediated immunity. The extent of TIL infiltration within the TME has been shown to correlate with the tumor’s mutational burden [36]. Notably, high TIL levels are associated with more aggressive clinicopathologic characteristics, such as higher cT stage and Ki-67 levels [37]. While high TIL levels are more commonly found in TNBC and Her-2-positive subtypes, they have been associated with pCR across all breast cancer subtypes. This is consistent with our findings, suggesting a uniform response of immunological infiltrates to chemotherapeutic agents, regardless of breast cancer subtype. Furthermore, high TIL levels (60% or more, consistent with our reporting) have been associated with improved prognosis in TNBC and Her-2-positive subtypes. However, in luminal Her-2-negative cancers, high TILs have been identified as a negative prognostic marker for overall survival [20]. This may be partially attributed to differences in the composition of immune cells within the infiltrate and it may indicate that, despite a favorable response to chemotherapy, other–possibly ER-related pathways–are contributing to the poor prognosis in this subgroup of luminal Her-2-negative cancers. Further research is needed to better understand both the role of peritumoral immune response and ER-related pathways in this subtype [36].

Beyond inflammatory cell infiltrates, TME consists of extracellular matrix (ECM), cancer-associated fibroblasts (CAFs), and vascular endothelial cells. Since fibroblasts in the stroma have both tumor-promoting and tumor-suppressing action, the role of CAFs has not yet been fully comprehended [38]. They are thought to promote angiogenesis, modulate the peritumoral immune response and restruct ECM components. They regulate cancer and other stromal cells, and potentially influence not only tumor growth and progression, but also modulate drug resistance mechanisms and immune evasion [18]. High stromal content, characterized by an abundance of CAFs, has been previously associated with poor response to both neoadjuvant and adjuvant therapy in breast and colorectal cancers [14, 18, 34].

Our findings are in line with the study of Li et al., who showed that a combination of TSR and TIL improves the prediction of ypT0/is ypN0 outcome (pCR in both breast and axilla), particularly in Her-2 negative tumors [39]. While responses to NAST in the breast and axilla often overlap [40], our study focused specifically on predicting nodal response, which is key in planning the axillary surgical procedure.

While it is not meant to replace the decision to opt for SLNB as initial surgical procedure in clinically negative axilla, an accurate nodal response prediction tool is beneficial in different borderline clinical scenarios. For example, when residual nodal disease is highly likely, SLNB procedure may be technically challenging with lower identification and higher false-negative rates [41]. In such cases, proceeding to ALND when in doubt, reduces the risk of potential undertreatment. Conversely, if there is a high probability of nodal pCR, and we attempt a limited surgical staging procedure (SLNB or targeted axillary dissection (TAD)), but fail to identify a minimum of three lymph nodes or targeted node, we can opt for ALND omission. This is because we are already aware of the high probability of nodal pCR.

The EUBREAST-01 trial aims to clarify the question of omitting axillary surgery in Her-2-positive and triple-negative patients who achieve a radiologic and pathologic complete response in the breast, but this is currently limited to cN0 patients. In cN+ patients, axillary surgical staging remains essential, as residual nodal disease reflects the chemoresistance of the disease and provides insights for further systemic treatment planning.

The current prediction model may have to be adjusted in the future to better recognize subgroups of patients that despite not achieving nodal pCR will be candidates for ALND omission (e.g., OPBC-07 Study), but according to current guidelines, omission of ALND is of now only advised within clinical studies (Alliance A01120202, ADARNAT, OPBC-03 Trials). The potential advantage of the model is also to help guide adjuvant RT/systemic treatment decisions in these cases.

Including radiologic assessment of response to NAST in the breast and axilla in the model would likely further improve the prediction tool. This has been previously noted by Kantor et al. who suggested that response in the breast is associated with response in the nodes, whether clinical, radiological or pathological [42]. However, in our cohort of 437 patients, only 45% underwent MRI limiting the inclusion of MRI assessment in the model. Nonetheless, MRI and other imaging modalities open the door to radiomics, which is becoming increasingly popular in cancer imaging analysis and could also be used in the post-NAST setting to assess nodal response [43].

The study has several limitations. The retrospective nature of the analysis harbors potential pitfalls. The stromal content from CBs was assessed by a single pathologist, reducing inter-observer variability but potentially introducing bias. The study spans over a relatively long period of time during which NAST regimens, as well as indications for NAST have changed. This most likely resulted in improved pCR rates over time. The trend may further continue (e.g., immunotherapy in the neoadjuvant setting for triple-negative cancers improves pCR rates). One fifth of the patients in our cohort had SLNB only as staging procedure, of which 75% were ypN0. While we aim to minimize the false-negative rates by dual-tracer technique and sampling of at least 3 SLNs, SLNB is not as accurate method of axillary staging as ALND. As a consequence, the proportion of nodal pCR in our study cohort may have been slightly overestimated. Nevertheless, it would not be acceptable to perform ALND for the sole purpose of axillary staging. Our data analysis was performed using machine learning techniques rather than traditional statistical approaches and lacks conventional null hypothesis testing. Although cross-validation ensures reproducibility of the model and improves generalizability, the most significant limitation of our study remains the lack of external validation [44].While our findings align with previous literature on predicting pCR, which enhances current study’s relevance and significance, we acknowledge that without validation of the model on an independent external cohort it is impossible to generalize our findings. Specifically, we aim to collaborate with other institutions within the EUBREAST Network to obtain external data sets, which will allow us to assess the robustness and applicability of our findings across different populations. We also encourage other centers to use similar tools on their own data to provide accurate risk estimation tools for individual patients and ultimately improve the outcome and cost-effectiveness of care [45].

## Conclusions

In summary, we have developed a clinical tool to predict nodal pCR for cN+ patients after NAST that includes biomarkers of TME and achieves an AUC of 0.86 after tenfold cross-validation. Similar tools are needed and we encourage the use of data mining techniques in clinical practice to improve outcomes.

## Supplementary Information

Below is the link to the electronic supplementary material.

Supplementary file1 (DOCX 23 KB)

Supplementary file2 (JPG 53 KB)

## Data Availability

The datasets generated and analysed during the current study are available from the corresponding author on reasonable request. A copy of visual programming workflow is available as a Supplement material. No datasets were generated or analysed during the current study.

## References

[1] Morrow M, Khan AJ (2020) Locoregional management after neoadjuvant chemotherapy. J Clin Oncol 38(20):2281–2291 32442069 10.1200/JCO.19.02576 PMC7343435

[2] Valachis A, Mamounas EP, Mittendorf EA et al (2018) Risk factors for locoregional disease recurrence after breast-conserving therapy in patients with breast cancer treated with neoadjuvant chemotherapy: an international collaboration and individual patient meta-analysis. Cancer 124(14):2923–2930 29723396 10.1002/cncr.31518

[3] Nguyen TT, Hoskin TL, Day CN et al (2018) Decreasing use of axillary dissection in node-positive breast cancer patients treated with neoadjuvant chemotherapy. Ann Surg Oncol 25(9):2596–2602 29978369 10.1245/s10434-018-6637-9

[4] Kuehn T, Bauerfeind I, Fehm T et al (2013) Sentinel-lymph-node biopsy in patients with breast cancer before and after neoadjuvant chemotherapy (SENTINA): a prospective, multicentre cohort study. Lancet Oncol 14(7):609–618 23683750 10.1016/S1470-2045(13)70166-9

[5] Boughey JC, Suman VJ, Mittendorf EA et al (2013) Sentinel lymph node surgery after neoadjuvant chemotherapy in patients with node-positive breast cancer: the ACOSOG Z1071 (alliance) clinical trial. JAMA 310(14):1455–1461 24101169 10.1001/jama.2013.278932 PMC4075763

[6] Boileau JF, Poirier B, Basik M, Holloway CMB, Gaboury L, Sideris L et al (2015) Sentinel node biopsy after neoadjuvant chemotherapy in biopsy-proven node-positive breast cancer: the SN FNAC study. J Clin Oncol 33(3):258–263 25452445 10.1200/JCO.2014.55.7827

[7] Caudle AS, Yang WT, Krishnamurthy S, Mittendorf EA, Black DM, Gilcrease MZ et al (2016) Improved axillary evaluation following neoadjuvant therapy for patients with node-positive breast cancer using selective evaluation of clipped nodes: implementation of targeted axillary dissection. J Clin Oncol 34(10):1072–1078 26811528 10.1200/JCO.2015.64.0094 PMC4933133

[8] Kahler-Ribeiro-Fontana S, Pagan E, Magnoni F, Vicini E, Morigi C, Corso G et al (2021) Long-term standard sentinel node biopsy after neoadjuvant treatment in breast cancer: a single institution ten-year follow-up. Eur J Surg Oncol 47(4):804–812 33092968 10.1016/j.ejso.2020.10.014

[9] Mamtani A, Barrio AV, King TA, Van Zee KJ, Plitas G, Pilewskie M et al (2016) How often does neoadjuvant chemotherapy avoid axillary dissection in patients with histologically confirmed nodal metastases? Results of a prospective study. Ann Surg Oncol 23(11):3467–3474 27160528 10.1245/s10434-016-5246-8 PMC5070651

[10] Banys-Paluchowski M, Gruber IV, Hartkopf A, Paluchowski P, Krawczyk N, Marx M et al (2020) Axillary ultrasound for prediction of response to neoadjuvant therapy in the context of surgical strategies to axillary dissection in primary breast cancer: a systematic review of the current literature. Arch Gynaecol Obstet 301:341–353 10.1007/s00404-019-05428-x 31897672

[11] Di Micco R, Zuber V, Fiacco E, Carriero F, Gattuso MI, Nazzaro L et al (2019) Sentinel node biopsy after primary systemic therapy in node positive breast cancer patients: time trend, imaging staging power and nodal downstaging according to molecular subtype. Eur J Surg Oncol 45(6):969–975 30744944 10.1016/j.ejso.2019.01.219

[12] Samiei S, Simons JM, Engelen SME, Beets-Tan RGH, Classe JM, Smidt ML (2021) Axillary pathologic complete response after neoadjuvant systemic therapy by breast cancer subtype in patients with initially clinically node-positive disease. JAMA Surg 156(6):e210891 33881478 10.1001/jamasurg.2021.0891 PMC8060891

[13] Ladak F, Chua N, Lesniak D, Ghosh S, Wiebe E, Yakimetz W et al (2022) Predictors of axillary node response in node-positive patients undergoing neoadjuvant chemotherapy for breast cancer. Can J Surg 65(1):89–96 10.1503/cjs.012920 PMC8834246 35135785

[14] Ravensbergen CJ, Polack M, Roelands J, Crobach S, Putter H, Gelderblom H et al (2021) Combined assessment of the tumor–Stroma ratio and tumor immune cell infiltrate for immune checkpoint inhibitor therapy response prediction in colon cancer. Cells 10(11):2935 34831157 10.3390/cells10112935 PMC8616493

[15] Kemi N, Eskuri M, Herva A, Leppänen J, Huhta H, Helminen O et al (2018) Tumour-stroma ratio and prognosis in gastric adenocarcinoma. Br J Cancer 119(4):435–439 30057407 10.1038/s41416-018-0202-y PMC6133938

[16] Wang K, Ma W, Wang J, Yu L, Zhang X, Wang Z et al (2012) Tumor-stroma ratio is an independent predictor for survival in esophageal squamous cell carcinoma. J Thor Oncol 7(9):1457–1461 10.1097/JTO.0b013e318260dfe8 22843085

[17] Jin HY, Yoo SY, Lee JA, Wen X, Kim Y, Park HE et al (2022) Combinatory statuses of tumor stromal percentage and tumor infiltrating lymphocytes as prognostic factors in stage III colorectal cancers. J Gastroenter Hepatol 37(3):551–557 10.1111/jgh.15774 35018665

[18] Alexander PG, Roseweir AK, Pennel KAF, van Wyk HC, Powell AGMT, McMillan DC et al (2021) The Glasgow Microenvironment Score associates with prognosis and adjuvant chemotherapy response in colorectal cancer. Br J Cancer 124(4):786–796 33223535 10.1038/s41416-020-01168-x PMC7884404

[19] Agarwal G, Vishvak Chanthar KMM, Katiyar S, Kumari N, Krishnani N, Sabaretnam M et al (2023) Predictive and prognostic role of tumor-infiltrating lymphocytes in patients with advanced breast cancer treated with primary systemic therapy. World J Surg 47(5):1238–1246 36735048 10.1007/s00268-023-06912-x

[20] Denkert C, von Minckwitz G, Darb-Esfahani S, Lederer B, Heppner BI, Weber KE et al (2018) Tumour-infiltrating lymphocytes and prognosis in different subtypes of breast cancer: a pooled analysis of 3771 patients treated with neoadjuvant therapy. Lancet Oncol 19(1):40–50 29233559 10.1016/S1470-2045(17)30904-X

[21] Kramer CJH, Vangangelt KMH, van Pelt GW, Dekker TJA, Tollenaar RAEM, Mesker WE (2019) The prognostic value of tumour–stroma ratio in primary breast cancer with special attention to triple-negative tumours: a review. Breast Cancer Res Treat 173:55–64 30302588 10.1007/s10549-018-4987-4 PMC6394568

[22] Dekker TJA, Van De Velde CJH, Van Pelt GW, Kroep JR, Julien JP, Smit VTHBM et al (2013) Prognostic significance of the tumor-stroma ratio: validation study in node-negative premenopausal breast cancer patients from the EORTC perioperative chemotherapy (POP) trial (10854). Breast Cancer Res Treat 139(2):371–379 23709090 10.1007/s10549-013-2571-5

[23] Pislar N, Gasljevic G, Ratosa I, Kovac A, Zgajnar J, Perhavec A (2023) Absence of post-treatment changes in sentinel lymph nodes does not translate into increased regional recurrence rate in initially node-positive breast cancer patients. Breast Cancer Res Treat 202:443–450 37679645 10.1007/s10549-023-07084-x PMC10564834

[24] Hagenaars SC, Vangangelt KMH, Van Pelt GW, Karancsi Z, Tollenaar RAEM, Green AR et al (2022) Standardization of the tumor-stroma ratio scoring method for breast cancer research. Breast Cancer Res Treat 193:545–553 35429321 10.1007/s10549-022-06587-3 PMC9114083

[25] Le MK, Odate T, Kawai M, Oishi N, Kondo T (2023) Investigating the role of core needle biopsy in evaluating tumor-stroma ratio (TSR) of invasive breast cancer: a retrospective study. Breast Cancer Res Treat 197(1):113–121 36335529 10.1007/s10549-022-06768-0

[26] Cha YJ, Ahn SG, Bae SJ, Yoon CI, Seo J, Jung WH et al (2018) Comparison of tumor-infiltrating lymphocytes of breast cancer in core needle biopsies and resected specimens: a retrospective analysis. Breast Cancer Res Treat 171(2):295–302 29869774 10.1007/s10549-018-4842-7

[27] Demšar J, Erjavec A, Hočevar T, Milutinovič M, Možina M, Toplak M et al (2013) Orange: data mining toolbox in python. J Mach Learn Res 14:2349–2353

[28] Možina M, Demšar J, Kattan M, Zupan B (2004) Nomograms for visualization of Naive Bayesian classifier. LNAI 3202:337–348

[29] McShane LM, Altman DG, Sauerbrei W, Taube SE, Gion M, Clark GM (2005) REporting recommendations for tumour MARKer prognostic studies (REMARK). Br J Cancer 93(4):387–391 16106245 10.1038/sj.bjc.6602678 PMC2361579

[30] Corsi F, Albasini S, Sorrentino L, Armatura G, Carolla C, Chiappa C et al (2021) Development of a novel nomogram-based online tool to predict axillary status after neoadjuvant chemotherapy in cN+ breast cancer: a multicentre study on 1,950 patients. Breast 60:131–137 34624755 10.1016/j.breast.2021.09.013 PMC8503563

[31] Guo R, Su Y, Si J, Xue J, Yang B, Zhang Q et al (2020) A nomogram for predicting axillary pathologic complete response in hormone receptor–positive breast cancer with cytologically proven axillary lymph node metastases. Cancer 126(S16):3819–3829 32710664 10.1002/cncr.32830

[32] Kim JY, Park HS, Kim S, Ryu J, Park S, Il KS (2015) Prognostic nomogram for prediction of axillary pathologic complete response after neoadjuvant chemotherapy in cytologically proven node-positive breast cancer. Medicine 94(43):e1720 26512562 10.1097/MD.0000000000001720 PMC4985376

[33] Richter AN, Khoshgoftaar TM (2018) A review of statistical and machine learning methods for modeling cancer risk using structured clinical data. Artif Intell Med 90:1–14 30017512 10.1016/j.artmed.2018.06.002

[34] Hagenaars SC, de Groot S, Cohen D, Dekker TJA, Charehbili A, Meershoek-Klein Kranenbarg E et al (2021) Tumor-stroma ratio is associated with Miller-Payne score and pathological response to neoadjuvant chemotherapy in HER2-negative early breast cancer. Int J Cancer 149(5):1181–1188 34043821 10.1002/ijc.33700 PMC8362217

[35] Albusayli R, Graham JD, Pathmanathan N, Shaban M, Raza SEA, Minhas F, et al (2023) Artificial intelligence-based digital scores of stromal tumour-infiltrating lymphocytes and tumour-associated stroma predict disease-specific survival in triplenegative breast cancer. J Pathol 260(1):32–42 10.1002/path.6061 36705810

[36] El Bairi K, Haynes H, Blackley E, Fineberg S, Shear J, Turner S et al (2021) The tale of TILs in breast cancer: a report from The International Immuno-Oncology Biomarker Working Group. NPJ Breast Cancer 7:150 34853355 10.1038/s41523-021-00346-1 PMC8636568

[37] Hwan H, Jung H, Hyeon J, Park Y, Ahn J, Im Y et al (2019) A nomogram to predict pathologic complete respone (pCR) and the value of tumor-infiltrating lymphocytes (TILs) for prediction of response to neoadjuvant chemotherapy (NAC) in breast cancer patients. Breast Cancer Res Treat 173(2):255–266 30324273 10.1007/s10549-018-4981-x

[38] Chen Y, McAndrews KM, Kalluri R (2021) Clinical and therapeutic relevance of cancer-associated fibroblasts. Nat Rev Clin Oncol 18(12):792–804 34489603 10.1038/s41571-021-00546-5 PMC8791784

[39] Li F, Chen H, Lu X, Wei Y, Zhao Y, Fu J et al (2023) Combining the tumor-stroma ratio with tumor-infiltrating lymphocytes improves the prediction of pathological complete response in breast cancer patients. Breast Cancer Res Treat 202(1):173–183 37528265 10.1007/s10549-023-07026-7

[40] Samiei S, Van Nijnatten TJA, De Munck L, Keymeulen KBMI, Simons JM, Kooreman LFS et al (2020) Correlation between pathologic complete response in the breast and absence of axillary lymph node metastases after neoadjuvant systemic therapy. Ann Surg 271(3):574–580 30557203 10.1097/SLA.0000000000003126

[41] Laws A, Hughes ME, Hu J, Barry WT, Dominici L, Nakhlis F et al (2019) Impact of residual nodal disease burden on technical outcomes of sentinel lymph node biopsy for node-positive (cN1) breast cancer patients treated with neoadjuvant chemotherapy. Ann Surg Oncol 26(12):3846–3855 31222687 10.1245/s10434-019-07515-4

[42] Kantor O, Sipsy LMN, Yao K, James TA (2018) A predictive model for axillary node pathologic complete response after neoadjuvant chemotherapy for breast cancer. Ann Surg Oncol 25(5):1304–1311 29368152 10.1245/s10434-018-6345-5

[43] Sutton EJ, Onishi N, Fehr DA, Dashevsky BZ, Sadinski M, Pinker K et al (2020) A machine learning model that classifies breast cancer pathologic complete response on MRI post-neoadjuvant chemotherapy. Breast Cancer Res 22(1):1–11 10.1186/s13058-020-01291-w PMC7254668 32466777

[44] Hunter DJ, Holmes C (2023) Where medical statistics meets artificial intelligence. N Engl J Med 389(13):1211–1219 37754286 10.1056/NEJMra2212850

[45] Shipe ME, Deppen SA, Farjah F, Grogan EL (2019) Developing prediction models for clinical use using logistic regression: An overview. J Thorac Dis 11:574–584 10.21037/jtd.2019.01.25 PMC6465431 31032076

