# Supplementary figures and images for: Predicting nodal response to neoadjuvant treatment in breast cancer with core biopsy biomarkers of tumor microenvironment using data mining
Source: Breast Cancer Res Treat. 2024 Nov 4;210(1):87–94. doi: 10.1007/s10549-024-07539-9 (PMC11787214; doi:10.1007/s10549-024-07539-9)

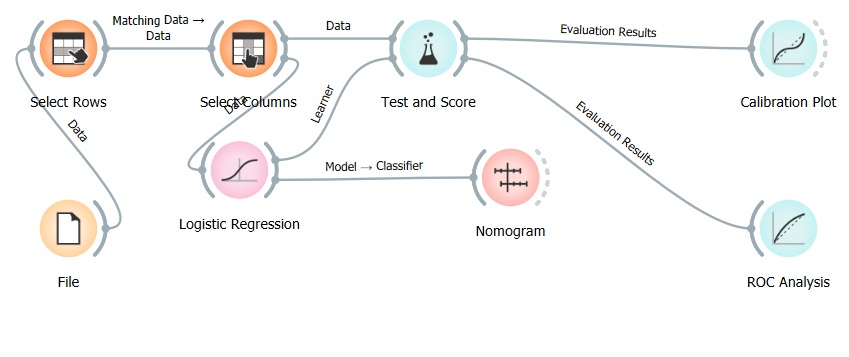

Supplement: Supplementary file 2 — Supplementary file2 (JPG 53 KB) [file 10549_2024_7539_MOESM2_ESM.jpg]
